# Atypical Carcinoid With Pulmonary Metastasis in an Adolescent

**DOI:** 10.1016/j.atssr.2024.05.023

**Published:** 2024-06-20

**Authors:** Ryo Karita, Hironobu Wada, Yuki Onozato, Toshiko Kamata, Hajime Tamura, Takashi Anayama, Mina Komuta, Yuichiro Hayashi, Ichiro Yoshino, Shigetoshi Yoshida

**Affiliations:** 1 Department of Thoracic Surgery, International University of Health and Welfare Narita Hospital, Narita, Japan; 2 Department of Pathology, International University of Health and Welfare Narita Hospital, Narita, Japan

## Abstract

Advanced-stage atypical carcinoid tumors are seldom seen in the teenaged population. Comprehensive care, extending beyond mere cancer treatment, is essential. A 16-year-old boy received a diagnosis of a 13-mm nodule in the left S^5^ lung segment with signs suggesting interlobar pleural indentation. A surgical biopsy revealed a neuroendocrine tumor, which led to lingular segmentectomy and lymph node dissection. The pathologic diagnosis was atypical carcinoid with intrapulmonary metastasis, classified as pT3 N0 M0 stage IIB. In addition to oncologic management for the advanced-stage atypical carcinoid, genetic counseling and meticulous mental support were provided. The accumulation of clinical data on teenaged patients with lung cancer is urgently needed.

Atypical carcinoids comprise a rare subtype of neuroendocrine tumors originating in the lungs. They account for 1% to 2% of malignant lung tumors, and are typically observed in younger patients.^1^ The occurrence of advanced-stage atypical carcinoids in teenagers is rare, and there is no established standard protocol. This report presents a case of an atypical carcinoid with pulmonary metastasis in a teenaged patient.

A 16-year-old boy was referred to our department (Department of Thoracic Surgery, International University of Health and Welfare Narita Hospital, Chiba, Japan) for further investigation and management of an abnormal shadow on chest radiography during a school checkup at the beginning of high school. There was no significant medical history, comorbidity, or medication use. The patient had no history of smoking. A chest computed tomographic (CT) scan showed a 13-mm nodule in the left S^5^ adjacent to the visceral pleura, with signs suggesting interlobar pleural indentation (Figure 1). Blood tests, including those for tumor markers, showed no abnormalities. Considering the patient’s age, a hamartoma was initially suspected; however, malignancy could not be ruled out on the basis of the CT findings. We decided to proceed with a surgical biopsy.

**Figure 1:**
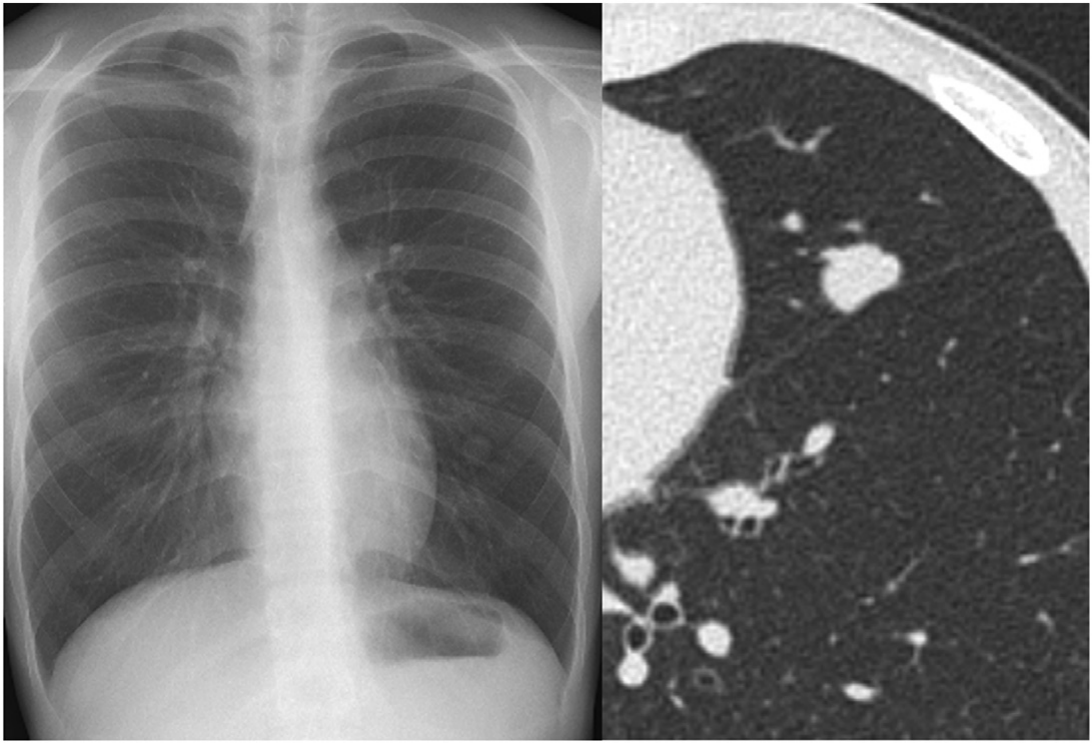
(Left) The chest roentgenogram showed an abnormal shadow in the left lower lung field. (Right) The computed tomographic image showed a 13-mm nodular shadow with suspected interlobar pleural indentation.

Thoracoscopy revealed a tumor with pleural indentation and vascular involvement (Figure 2). Pleural effusion and dissemination were not observed. Wedge resection was performed, and an intraoperative pathologic examination suggested a neuroendocrine tumor, thus raising the possibility of small cell carcinoma in some areas. Left lingular segmentectomy with lymph node dissection was subsequently performed with curative intent. The postoperative course was uneventful, and the patient was discharged home 4 days after surgery.

**Figure 2:**
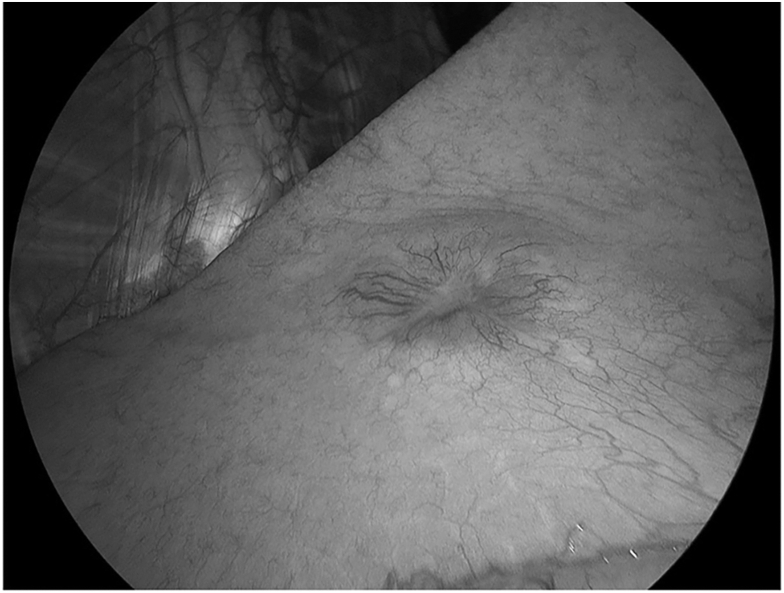
The tumor had pleural indentation and dilated capillaries.

Hematoxylin and eosin staining revealed a well-defined solid tumor adjacent to the pleura with distinctive boundaries. The tumor cells, characterized by round nuclei, formed nests and exhibited rosette patterns. Additionally, focal cancer growth involving lymphatic invasion was identified apart from the primary lesion, resulting in a diagnosis of PM1. Eight mitotic figures were observed in 10 high-power fields (2 mm^2^), and there was no apparent necrosis. Results of immunohistochemical staining were negative for thyroid transcription factor 1 but were diffusely and strongly positive for CD56, synaptophysin, and chromogranin. D2-40 staining confirmed the presence of lymphatic invasion (Figure 3). The final pathologic diagnosis was atypical carcinoid pT3 N0 M0 stage IIB.

**Figure 3:**
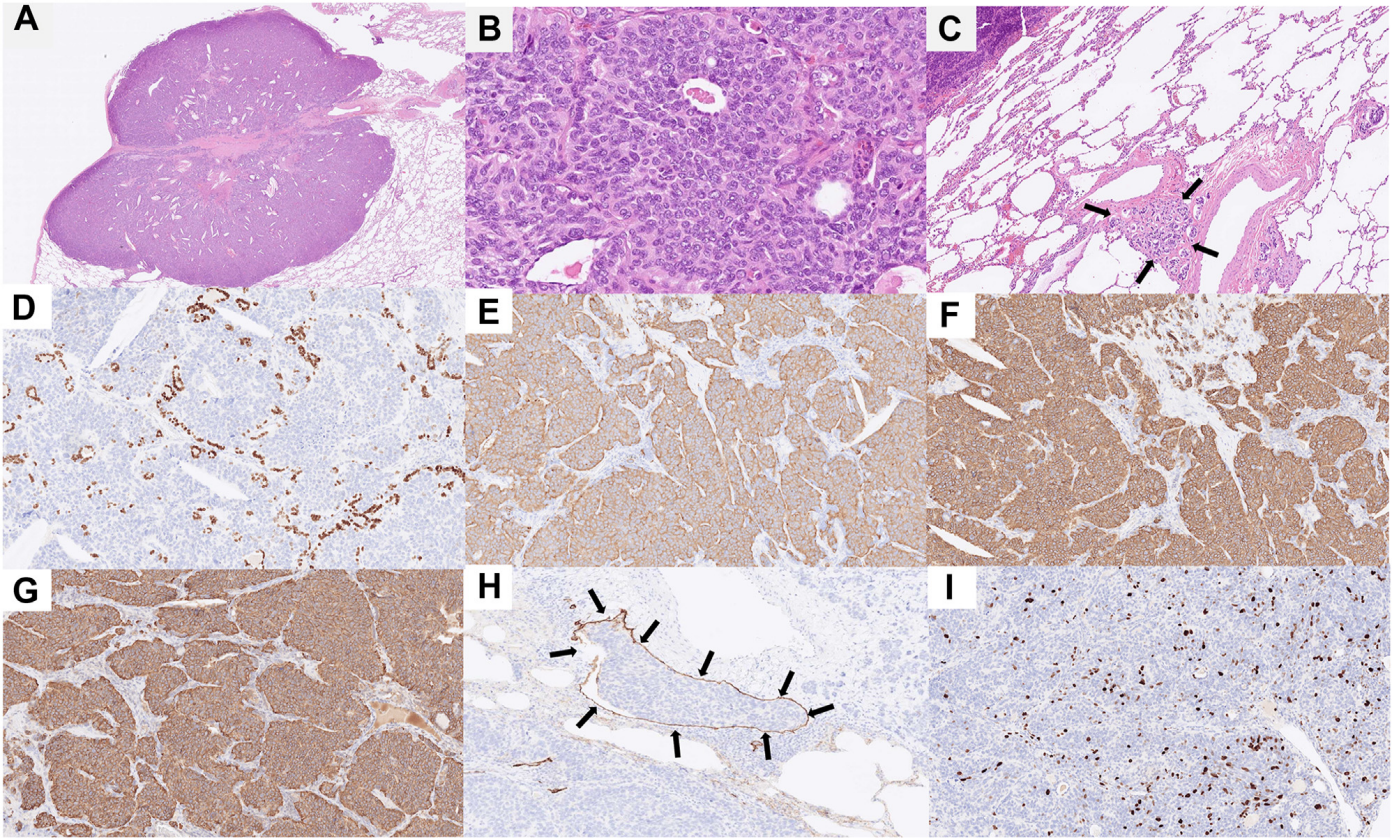
(a) Hematoxylin and eosin staining shows a clearly demarcated, enhanced tumor. (b) Tumor cells with round nuclei are proliferating. (c) Cancerous growth by lymphatic invasion (arrows). (d) Results of immunohistochemical staining were negative for thyroid transcription factor 1. The brown-stained cells are nonneoplastic alveolar epithelium left behind within the tumor. (e-g) Results of immunohistochemical staining are positive for (e) CD56, (f) synaptophysin, and (g) chromogranin. (h) D2-40 staining shows lymphatic invasion (arrows). (i) The Ki-67 index at the hot spot is 8%.

Postoperatively, fluorine-18 2-fluoro-2-deoxy-D-glucose-positron emission tomography–CT imaging confirmed the absence of distant metastasis. Contrast-enhanced magnetic resonance imaging of the head ruled out brain metastases and pituitary adenomas. Contrast-enhanced whole-body CT ruled out thyroid and adrenal tumors, and upper and lower gastrointestinal endoscopy revealed no tumors in the digestive tract, thus negating the potential for multiple endocrine neoplasia, which is known to be associated with carcinoid tumors. The surgical specimen was subjected to genetic testing, which was performed using the Oncomine Dx Target Test Multi CDx System (Thermo Fisher Scientific) and immunohistochemical staining for PD-L1 (22C3, Agilent Technologies). These tests revealed no genetic mutations, and the PD-L1 tumor proportion score was 2%.

Considering the advanced-stage atypical carcinoid, adjuvant chemotherapy was considered as a treatment option. However, evidence on adjuvant chemotherapy for atypical carcinoid in teenagers is scarce, and the potential impact of chemotherapy on the reproductive function and quality of life are well known. The disease stage, prognosis, and potential side effects of chemotherapy were meticulously discussed, and genetic counseling was provided. Ultimately, the patient and his mother opted for close observation, without further treatment. Currently, the patient remains free of recurrence at 8 months after surgery.

## Comment

This report describes a remarkably rare case of atypical carcinoid with pulmonary metastasis in a teenaged patient. The 2021 Japanese annual registry reported only 20 instances of malignant lung tumors among patients aged <20 years from a total of 46,589 cases (0.04%), a finding indicating its extreme rarity. In the same data set, carcinoid tumors accounted for 226 of 47,029 lung tumor surgical procedures (0.48%), a number highlighting their uncommon occurrence.^2^

The first choice for the local treatment of atypical carcinoids is surgical intervention.^1^ Anatomic lung resections with lymph node dissection are recommended procedures, whereas a recent analysis demonstrated no significant difference in overall survival between lobectomy and sublobar resection in patients with atypical carcinoid of the lung.^3^ A nationwide retrospective study showed 5-year overall survival after surgical resection for stage I, II, III, and IV atypical carcinoids of 84%, 74%, 52%, and 51%, respectively.^4^ The European Society of Medical Oncology stated that the recurrence of atypical carcinoid was observed in up to 35% of patients, with approximately one-third having local recurrences.^5^ Surgical resection with curative intent is recommended for local recurrence when technically feasible, whereas everolimus is recommended for unresectable or advanced atypical carcinoids.^5^

There is no definitive evidence to support the survival benefits of postoperative adjuvant chemotherapy for typical or atypical carcinoids.^1^ A large-scale retrospective analysis reported no superiority in the 5-year overall survival of postoperative adjuvant chemotherapy to observation in patients with stage II atypical carcinoids.^6^ Guidelines from various organizations, including the National Comprehensive Cancer Network, European Neuroendocrine Tumor Society, and European Society of Medical Oncology, recommend surgical resection alone without adjuvant chemotherapy for patients with stage I or II typical and atypical carcinoids.^7^

Teenagers require comprehensive management because they primarily focus on their school life. Treatment-related long-term absences and changes in appearance related to chemotherapy should be considered because they may affect a patient’s mental status and education. When considering chemotherapy for teenaged patients, preserving fertility and the reproductive function is crucial. Psychological management should also be provided. Pediatricians, psychiatrists, oncology-certified nurses, and other health care professionals should be involved. For teenagers with lung cancer, there are numerous challenges to consider beyond oncologic issues. Despite their complexity, the limited number of cases and insufficient information remain the primary problems. To address these challenges in the future, it is essential to accumulate more cases and share information.

In conclusion, we report a case of an advanced-stage atypical carcinoid in a teenager. The accumulation of clinical data from similar cases will aid clinicians in making appropriate decisions.
